# The role of dephasing for dark state coupling in a molecular Tavis–Cummings model

**DOI:** 10.1063/5.0155302

**Published:** 2023-07-28

**Authors:** Eric Davidsson, Markus Kowalewski

**Affiliations:** Department of Physics, Stockholm University, Albanova University Center, SE-106 91 Stockholm, Sweden

## Abstract

The collective coupling of an ensemble of molecules to a light field is commonly described by the Tavis–Cummings model. This model includes numerous eigenstates that are optically decoupled from the optically bright polariton states. Accessing these dark states requires breaking the symmetry in the corresponding Hamiltonian. In this paper, we investigate the influence of non-unitary processes on the dark state dynamics in the molecular Tavis–Cummings model. The system is modeled with a Lindblad equation that includes pure dephasing, as it would be caused by weak interactions with an environment, and photon decay. Our simulations show that the rate of pure dephasing, as well as the number of two-level systems, has a significant influence on the dark state population.

## I Introduction

Polaritonic chemistry—where chemical reactions are studied in the presence of a strongly coupled electromagnetic field—is continuing to attract interest in fields ranging from chemistry to quantum optics, as shown by recent review studies.^1–7^ These systems have been explored in what is becoming a rich landscape of different experiments. Examples include demonstrating single-molecule strong coupling,^8^ investigating vibrational strong coupling,^9–11^ strong coupling in J-aggregates,^12^ photo-isomerization,^13,14^ and two-dimensional spectroscopy of polaritonic systems.^15,16^ Along with experiments, the community has also made significant theoretical advancements. Among the investigated phenomena, we can highlight studies of conical intersections,^17–19^ photon up-conversion,^20^ polaritonic spectra,^21,22^ photo-dissociation,^23,24^ and model refinements from the Cavity–Born–Oppenheimer approximation.^25,26^

One of many challenges for this developing field is to build models that incorporate the details relevant to chemical reactivity (e.g., molecular vibrations along reaction coordinates) together with collective ensemble effects (several molecules coupled to the cavity field).^27,28^ Thus, in the last decade, several studies have turned their attention to such models of emitter ensembles.^24,27,29,30^

**Fig. 1:**
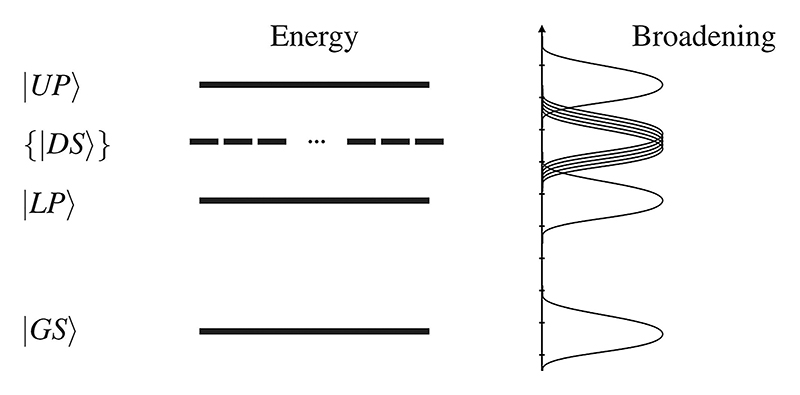
Schematic representation of the Tavis–Cummings model. The upper ∣*UP*⟩ and lower polariton states ∣*LP*⟩ are split by a collective Rabi frequency. The *N* − 1 dark states ∣*DS*⟩ do not couple to the electromagnetic field and are, thus, unaffected by the cavity. Line broadening, caused by dephasing, for example, introduces a coupling of the dark states with the polariton states.

In these ensemble models, a common feature is the presence of dark states, which do not couple to the field of the cavity or an external field. These states are most straightforward to consider in a Tavis–Cummings model limited to a single excitation:^31^ with *N* identical emitters, there are two bright polaritonic states, ∣*UP*⟩ and ∣*LP*⟩, which are symmetrical under emitter permutation. The remaining *N* −1 polaritonic states are dark, see Fig. 1. In the Tavis–Cummings model, dark states are degenerate,^32^ asymmetrical under emitter permutation,^33,34^ have no transition dipole moment, and correspond to collective emitter excitations, which gain no population during Hamiltonian evolution.^24^ Note that these dark states emerge in the ensemble system, which is different from disallowed transitions in individual emitters.

The absence of population in the dark states makes intuitive sense; assuming an initial symmetrical state, the Hamiltonian treats all emitters identically, so unitary evolution cannot induce asymmetry. The existence of the dark states, as well as their properties, are also quite robust; for example, they persist even if the field couplings vary among emitters.^35^ In this work, the model will be an extended version of the Tavis–Cummings model, but properties of the dark states still hold (degeneracy, asymmetry, non-existent transition dipole moment, and zero population under Hamiltonian evolution).

Since the dark states are often assumed not to take part in the dynamics, they are commonly discarded from the deployed models. However, there are numerous publications discussing how these states are physically significant. They have been studied in the broader context of cavity QED,^32,36–40^ quantum thermodynamics,^41^ and polaritonic chemistry.^27,42–44^ In the latter context, these states are important both when the electromagnetic field couples to electronically excited states^17,45–48^ and vibrational states.^33,49–52^ Despite the theoretical focus of numerous studies, the presence of dark states can by no means disregarded in experimental studies.^15,53–59^

The role of dephasing processes and their influence on dark-state dynamics are intertwined with identifying a mechanism for their population.^48^ To lift the decoupling of the dark states from the bright polariton states, an asymmetry in the Hamiltonian (or the Liouvilian) is required. To find such a process, we can consider processes that operate at the level of individual emitters, such as the interactions with each emitter’s environment. We model this with dephasing operators, which can be included in the equations of motion by means of the Lindblad equation,

(1) $$\partial_{t}\hat{\rho }=-\frac{\text{i}}{\hbar }[\hat{H},\hat{\rho }]+\underset{n}{\sum \;}\left({\hat{L}}_{n}\hat{\rho }{\hat{L}}_{n}^{†}-\frac{1}{2}{\left[{\hat{L}}_{n}^{†}{\hat{L}}_{n},\hat{\rho }\right]}_{+}\right).$$

The emitter dephasing can be motivated by fluctuations in the relative energies between the states in the emitter systems^60^ or by a build-up of system–environment correlations.^61^

In the context of strongly coupled molecular systems, there has been a recent focus on the utility of modeling decay processes with the Lindblad equation.^23,62,63^ A consequence of using a density matrix based time evolution, however, is an increase in computational complexity. For the situation where only decay processes out of the Hilbert space under consideration are involved, an effective non-Hermitian Hamiltonian together with a Schrödinger equation can be used.^64–66^ However, when dephasing becomes relevant, such simplifications will be harder to make.

The quantum trajectories method (also called the stochastic Schrödinger equation^67^ or Monte Carlo wave function^68^ method) can be used to bypass the explicit evaluation of the density matrix. Here, we use this method to time-evolve the Lindblad equation using an ensemble of stochastic pure-state evolutions.^68,69^ Quantum trajectories are not yet widely used for polaritonic chemistry problems, but some studies have started to emerge.^70^

In this work, we investigate the role of dephasing for molecular strong coupling and its effects on the dark state dynamics. We include the effects of both dephasing and photon decay. The model is based on the Lindblad equation and implemented with quantum trajectories.

## II System And Model

The model system is based on an optical cavity with a single carbon monoxide molecule, where the fundamental cavity mode is resonant with a transition between the ^1^Σ^+^ ground-state and an ^1^Π electronically excited state in the CO molecule. To simulate a many molecule system with dark states, which is also resonant with the cavity mode, *N* two-level systems are added to the Hamiltonian. The Hamiltonian is, thus, an extension of the Tavis–Cummings model^4^ (an optical cavity with *N* two-level systems under the rotating wave approximation), where the extension is the CO molecule with its internuclear coordinate.

The Hamiltonian consists of the following five parts:

(2) $$\hat{H}={\hat{H}}_{c}+{\hat{H}}_{a}+{\hat{H}}_{ca}+{\hat{H}}_{m}+{\hat{H}}_{cm},$$

where *Ĥ*_*c*_ corresponds to a single fundamental mode of linearly polarized light in an optical cavity, *Ĥ*_*a*_ are the *N* two-level emitter systems (or atoms), *Ĥ*_*ca*_ are all the resonant cavity–atom interactions, *Ĥ*_*m*_ is the CO molecule, *Ĥ*_*cm*_ is the cavity–molecule interaction.

The cavity mode is modeled in the Fock basis {∣0⟩, ∣1⟩, ∣2⟩, …} and described by the following Hamiltonian:

(3) $${\hat{H}}_{c}=\hbar \omega_{c}{\hat{a}}^{†}\hat{a},$$

where *â*^†^ and *â* are the photon creation and annihilation operators, respectively, and *ω*_*c*_ is the cavity mode frequency. The cavity photon energy is fixed at *ħω*_*c*_ = 8.27 eV (*λ* = 150 nm), which makes the transition between electronic states in the CO molecule resonant with the cavity. This choice puts the minima of the two potentials at similar energies and prevents the population from accumulating in either well, see Fig. 3.

In addition to the CO molecule, the optical cavity contains *N* two-level emitters (or atoms), where *N* ∈ {0, 1, 2, 3, 5, 8, 13, 22, 36, 60} is a modulated parameter,

(4) $${\hat{H}}_{a}=\sum_{n=1}^{N}\hbar \omega_{a}{\hat{\sigma }}_{n}^{†}{\hat{\sigma }}_{n},\;$$

where ∣*g*_*n*_⟩ and ∣*e*_*n*_⟩ are the ground and excited states of the *n*th two-level system, respectively. The operators ${\hat{\sigma }}_{n}=|g_{n}\rangle \langle e_{n}|$ and ${\hat{\sigma }}_{n}^{†}=|e_{n}\rangle \langle g_{n}|$ de-excite and excite the *n*th two-level system, respectively. The energy of the transition is chosen to be resonant to the cavity’s photon energy, *ħω*_*a*_ = *ħω*_*c*_ = 8.27 eV.

The interaction between emitters and cavity mode is derived under the dipole and the rotating wave approximations,

(5) $${\hat{H}}_{ca}=\sum_{n=1}^{N}{ℰ}_{c}(N)\mu_{a}\left({\hat{a}}^{†}{\hat{\sigma }}_{n}+\hat{a}{\hat{\sigma }}_{n}^{†}\right)\;.$$

**Fig. 2:**
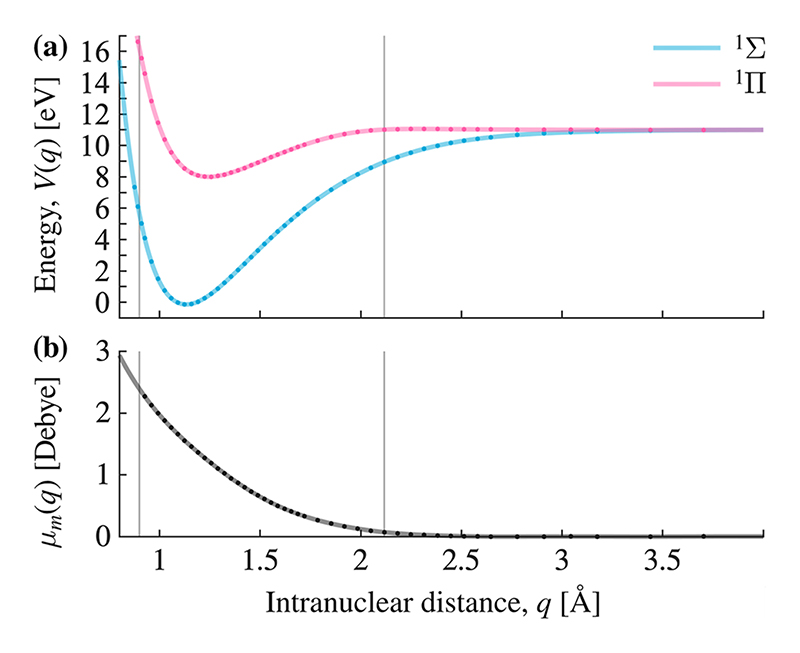
Potential energy curves and transition dipole curves for the CO molecule. Dots mark frozen internuclear distances from the quantum chemistry calculation. Vertical lines surround the region used in the time-evolution. (a) Potential energy surfaces for the ^1^Σ ground-state and one ^1^Π excited state. (b) Transition dipole moment between ^1^Σ and ^1^Π.

The transition dipole moments of all two-level systems, *μ*_*a*_, are identical and set to 1.5 Debye, which is on the same order of magnitude as the CO molecule [see Fig. 2(b)].

The CO molecule is modeled by including its electronic ^1^Σ ground-state potential energy curve, and one of the two electronically excited ^1^Π states, which has a non-zero transition dipole *μ* (*q*) moment perpendicular to the molecular axis, is assumed to be aligned with the field polarization. The molecular Hamiltonian reads

(6) $${\hat{H}}_{m}=-\frac{\hbar^{2}}{2M}\frac{{\text{d}}^{2}}{\text{d}q^{2}}+{\hat{V}}_{\text{Σ}}(q)|\text{Σ}\rangle \langle \text{Σ}\left|+{\hat{V}}_{\text{Π}}(q)\right|\text{Π}\rangle \langle \text{Π}|.$$

Here, the first term is the kinetic energy of the nuclei, with the reduced mass of the CO molecule (*M* = 12 498 *m_e_*). The potential energy curves for *V*_*Σ*_ (*q*) and *V*_*Π*_ (*q*) are shown in Fig. 2(a).

The previous choice of photon energy makes the molecular transition resonant with the cavity mode at an internuclear distance of *q* = 1.17 Å,

(7) $${\hat{H}}_{cm}={ℰ}_{c}(N)\mu_{m}(q)\left({\hat{a}}^{†}|\text{Σ}\rangle \langle \text{Π}|+\hat{a}|\text{Π}\rangle \langle \text{Σ}|\right).$$

The transition dipole moment $\mu_{m}(\hat{q})$ varies between about 0 and 3 Debye according to Fig. 2(b). The vacuum electric field strength, *ℰ*_*c*_(*N*), is the same as the atoms are experiencing [see Eq. (5)].

The vacuum electric field strength, *ℰ*_*c*_(*N*), in Eqs. (5) and (7), is fixed at 3.00 V/nm for a single CO molecule model with no atoms (i.e., *N* = 0). To compensate for an increase in collective coupling strength, as we increase the number of atoms in the model, the vacuum field strength is scaled by $1/\sqrt{N+1}$,^48^

(8) $${ℰ}_{c}(N)=\frac{3.00}{\sqrt{N+1}}\text{V}/\text{nm}$$

Note that the effects of the single molecule coupling strength can not be directly compared to a collective coupling strength of equal size. Thus, the results are expected to show a different behavior.^24^

The highest single molecule cavity coupling strength, *g*_avg_ = *ℰ*_*c*_*μ*_avg_, occurs for *N* = 0. We compare this to the typical energy scale, *g*_avg_/*ħω*_*c*_ = 0.011. The system operates well below the ultra-strong coupling regime. Note that we assumed an average transition dipole moment of *μ*_avg_ = 1.5 Debye.

In addition to the unitary part of the Hamiltonian, there are two non-unitary physical processes, which are introduced through the Lindblad Eq. (1). The Lindblad framework assumes that the system–bath correlations times are short enough to allow for the Markovian approximation.^49,71^ Thus, our results address interactions that can be approximated as Markovian.

The first non-unitary process is single-emitter dephasing of the CO molecule and all *N* two-level systems. The corresponding rate of dephasing, *γ*, is the key parameter whose effects we aim to investigate. It is the same for all emitters, which have their individual dephasing operator,

(9) $${\hat{L}}_{n}:=\sqrt{\frac{\gamma }{2}}{\hat{\sigma }}_{z}^{\left(n\right)},$$

where ${\hat{\sigma }}_{z}^{\left(n\right)}$ is the third Pauli matrix for the *n*th emitter,

(10) $${\hat{\sigma }}_{z}=\left[\begin{array}{cc} 1 & 0\\ 0 & -1 \end{array}\right].\;$$

Other choices of this operator yield the same time-evolution. However, this choice is beneficial for the quantum trajectory method since it does not transfer the population between states.^72^

When constructing the Lindblad dephasing operators, we neglect the fact that the sub-systems are coupled (by the cavity mode) and build them phenomenologically. This is generally considered acceptable outside the ultra-strong coupling regime.^73,74^ In the Appendix, we discuss the potential problems with this approach and argue that the phenomenological operators are appropriate.

The second non-unitary process is photon decay caused by a lossy cavity. The Lindblad operator for a single cavity mode, with the photon decay rate *κ*, reads

(11) $${\hat{L}}_{c}:=\sqrt{\kappa }\hat{a}.$$

The photon lifetime is fixed at *τ* = 100 fs, yielding *κ* = 1/*τ* = 0.01 fs^−1^. For the chosen photon energy at *ħω*_*c*_ = 8.27 eV, this corresponds to a quality factor of *Q* = 1.26 × 10^3^. For comparison, Q-factors reported in the literature range from 10^154,75^ for plasmonic nanoparticles^8^ to 10^4 75–77^ for Fabry–Pérot cavities made of Bragg reflectors.

## III Methods

Our studied observable is energy retention, i.e., we consider the fraction of the initial excitation that, despite the photon decay, remains in the system after 500 fs. The duration is chosen in relation to the timescale of nuclear dynamics and mirrors previous investigations.^24,62^ The initial excitation that consists of the cavity mode is in its first excited (single photon) state, while all emitters are in their ground-states. This limits the total number of excitations to one, which allows us to truncate the basis.

A direct solution of the Lindblad Eq. (1) with a density matrix scales quadratically with the number of states involved. Such an approach becomes prohibitive as the number of atoms in our system increases. Instead of modeling a statistical state as a single density operator, we use quantum trajectories^68,69^ to obtain the statistics from an ensemble of pure state wave functions. Thus, the cost is shifted from a single memory consuming density matrix to running multiple, but lighter, pure-state calculations with wave functions, {*Ψ*_*i*_ (*t*)}. Each wave function evolves stochastically, with “quantum jumps” occurring randomly in proportion to physical parameters. One can prove that in the limit of an infinite ensemble of wave functions, the state from evolution with quantum trajectories approaches the state from the Lindblad equation.^68^

From the ensemble of *N*_*T*_ trajectories, {*Ψ*_*i*_ (*t*) : *i* ∈ 1 … *N*_*T*_}, a density matrix can be recovered by summing outer products of each wave function with a uniform weight,

(12) $$\hat{\rho }=\frac{1}{N_{T}}\sum_{i=1}^{N_{T}}|\psi_{i}\rangle \langle \psi_{i}|\;$$

The respective expectation values {*Â_i_*⟩} can be obtained directly from the weighted sum of all trajectories, without having to construct $\hat{\rho }$ explicitly,

(13) $$\text{Tr}[\hat{A}\hat{\rho }]=\frac{1}{N_{T}}\sum_{i=1}^{N_{T}}\langle \hat{A}i\rangle \;$$

The quantum trajectory method requires two modifications to the time-dependent Schrödinger equation. The first one adds the last term from Eq. (1), i.e., $\sum_{n}-1/2\;{\left[{\hat{L}}_{n}^{†}{\hat{L}}_{n},\hat{\rho }\right]}_{+}.$, to the Hamiltonian in the form of a norm-decaying term. However, in our choice of implementation, the wave function is continuously renormalized (at each discrete time-step). Using the Lindblad operators from Eqs. (9) and (11) leads to the non-Hermitian Hamiltonian,

(14) $${\hat{H}}^{\prime }:=\hat{H}-\text{i}\hbar \frac{\kappa }{2}{\hat{a}}^{†}\hat{a}-\text{i}\hbar \frac{\gamma }{2}\sum \;n{\left({\hat{\sigma }}_{z}^{\left(n\right)}\right)}^{†}{\hat{\sigma }}_{z}^{\left(n\right)}.$$

The second term in Eq. (14) is responsible for the photon decay, while the third term only affects the norm of the total wave function since ${\left({\hat{\sigma }}_{z}^{\left(n\right)}\right)}^{†}{\hat{\sigma }}_{z}^{\left(n\right)}=\hat{𝟙}$. The algorithm used for the propagation renormalizes the wave function in each step, and thus, the last term from Eq. (14) has no effect and can be removed. The non-Hermitian modifications to the Hamiltonian are thus required for processes that cause a change in population, such as decay and pumping, but not for processes describing pure dephasing. A Hamiltonian, such as in Eq. (14), has been used in wave function calculations to describe photon decay.^64,66,78^ However, this part alone cannot capture the loss of phase information between the states involved, and using a wave-function-only is only valid when the coherence does not influence the states of interest.

The dephasing caused by photon decay and pure dephasing is captured by introducing random discrete stochastic jumps and combining multiple trajectories via Eq. (13). These jumps originate from the first term in Eq. (1), i.e.,$\sum_{n}{\hat{L}}_{n}\hat{\rho }{\hat{L}}_{n}^{†}\;$. The probability of a jump occurring during a time-interval Δ*t* depends on the rates *κ* and *γ* as well as the population in the subspace from which the jump occurs

(15) $$P\left(\;\text{jump with}\;{\hat{L}}_{k}\;\text{during}\;\text{Δ}t\right)=\text{Δ}t\langle \Psi (t)\left|{\hat{L}}_{k}^{†}{\hat{L}}_{k}\right|\Psi (t)\rangle .\;$$

Note here that the Lindblad operators *L*_*k*_ include the rates, *κ* and *γ*, as shown in Eqs. (8) and (11). At each time-step, random numbers are generated to determine if a jump occurs, in which case the Lindblad operator ${\hat{L}}_{k}$ is applied to the wave function, and the wave function is normalized.

We have implemented the quantum trajectories approach with our in-house software package QDng, which allows for the time evolution of wave functions. Implementations into existing methods are recurring in the literature.^70,72,79^ For each statistical state [see Eq. (12)], *N*_*T*_ = 2500 wave function trajectories were run. Estimates of the resulting errors are given in the caption of Fig. 4. Each wave function was time-evolved with the Arnoldi propagation method^80^ at an order of 10 and a time-step of 0.5 au (0.012 fs).

The time-evolution is carried out in a product basis composed of the field-free molecular states, the field free two-level system states, and the Fock states of the cavity mode. In the following, we will refer to this basis as a product basis.

**Fig. 3:**
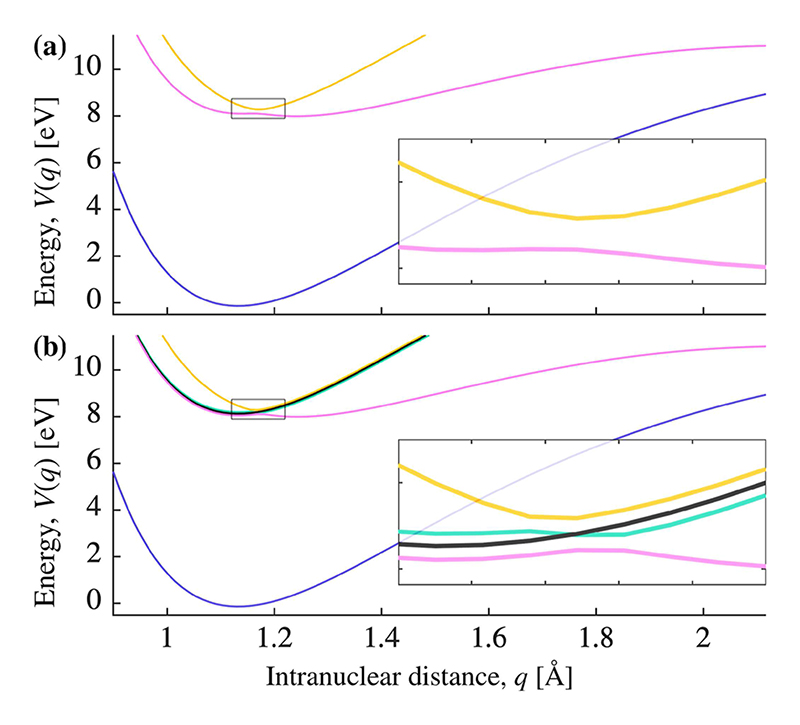
Potential energy curves of the polaritonic basis. (a) Polaritonic states for a single CO molecule and no two-level systems (*N* = 0), with the ground state (blue) and the upper (yellow) and lower (pink) polariton states. (b) Polaritonic states for a single CO molecule and two two-level systems (*N* = 2). Here, the first dark state is introduced (black) in addition to the middle polariton state (green). Note that the middle polariton state is a result of the nuclear degree of freedom of the CO molecule: it causes symmetry breaking, which lifts the degeneracy of one dark state. It is also dark when it crosses the other dark state, i.e., where the green and black curve cross. For higher values of *N* > 2, the curves are the same,^24^ but additional degenerate dark states are introduced.

For the purpose of interpreting the data (Sec. IV), we diagonalize the potential curves to obtain polaritonic curves (see Fig. 3). This can be achieved by a pointwise diagonalization of the Hamiltonian along the reaction coordinate, *q*, while omitting the kinetic energy operator. The number of polaritonic energy surfaces depends on *N*, but for *N* > 2, all additional energy surfaces are degenerate dark states. Thus, plotting the result for *N* = 0 and *N* = 2 gives an overview of all other values of *N* [see Fig. 3(b)]. Note that dark states are a feature of the polaritonic basis and do not appear in the product basis. We ensure that uncoupled polaritonic surfaces are allowed to cross by following the eigenvector tracking method described in Ref. 24.

The potential energy curves and dipole functions of the CO molecule, shown in Fig. 2, are the relevant results of a quantum chemistry calculation that originally included eight non-degenerate states and their respective transition dipole moments. The electronic structure calculations were carried out with the program package Molpro^81–83^ at the CASSCF(10/14)/MRCI/aug-cc-pVQZ level of theory, with a state average over a total of 12 electronic states. Energies, dipole moments, and transition dipole moments are calculated at 50 internuclear distances, between 0.926 and 6.35 Å. The two electronic states included in this work are ^1^Σ^+^, and one of the two doubly degenerate ^1^Π states. The data are in good agreement with previous calculations.^84,85^ The molecular potentials and transitions moments are interpolated to a spatial grid with 96 grid points in the interval 0.90 ≤ *q* ≤ 2.12 Å (see vertical lines in Fig. 2). The result is used to construct the molecular Hamiltonians, *Ĥ*_*m*_ and *Ĥ*_*cm*_ in Eqs. (6) and (7), for the numerical calculations.

## IV Results and Discussion

**Fig. 4:**
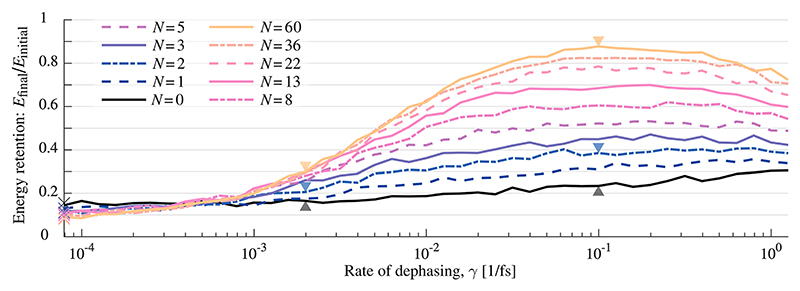
Energy retention in relation to dephasing rate. The vertical axis shows energy retention, i.e., the remaining energy as a fraction of initial energy, *E*_final_/*E*_initial_, where *E*_initial_ = *ħω*_*c*_, after 500 fs of time-evolution. The horizontal axis is the single emitter dephasing rate, *γ*, which is the same for all emitters. Each curve corresponds to a particular number of two-level systems, *N*. The number of dark states for each curve is *N* − 1. Crosses on the vertical axis show the energy retention for *γ* = 0. Arrows mark points where dynamics plots are supplied, see Figs. 5–7. Standard deviations due to the Trajectories method (from a set of 25 runs) are also calculated at these points. Gray arrows both have *σ* ≈ 0.008, left blue has *σ* ≈ 0.011, right blue has *σ* ≈ 0.012, left yellow has *σ* ≈ 0.009, and right yellow has *σ* ≈ 0.008.

To construct a molecular Tavis–Cummings model, which includes dark states, *N* two-level systems were introduced along with the CO molecule. We included the values *N* ∈ {0, 1, 2, 3, 5, 8, 13, 22, 36, 60}, and for each value, the dephasing rate is varied between 0% and 10% of the cavity frequency *ω*_*c*_, which corresponds to the dephasing rates: 0 ≤ *γ* ≤ 1.26 fs^−1^. The remaining energy in the system is then plotted as a fraction of the initial energy, in relation to the dephasing rate for each *N*, Here, we define the energy retention *R* as the ratio of the initial energy in the system and the remaining energy in the system at 500 fs,

(16) $$R=\frac{E(t=500\;\text{fs})}{E(t=0\;\text{fs})}=\frac{{\langle \hat{H}\rangle }_{t=500\;\text{fs}}}{{\langle \hat{H}\rangle }_{t=0\;\text{fs}}},$$

where *Ĥ* is the full Hamiltonian from Eq. (2). This constitutes the main result in this study and is shown in Fig. 4. Note that the obtained curves are not perfectly smooth, which is an expected result of the stochastic sampling of the density matrix.

Only 10%–15% of the initial energy is retained in the system for the dephasing free case (*γ* = 0, see crosses on the vertical axis in Fig. 4). With no dephasing, the *N* − 1 dark states are not populated and do not impact the behavior of the system. However, even though the number of bright states is fixed and with a constant collective coupling strength [see Eq. (8)], the energy retention for *γ* = 0 varies. This can be explained as follows: the constant collective Rabi splitting appears to behave similarly to the single molecule Rabi splitting between the upper and lower polaritons (see Fig. 3). However, the splitting between the middle polariton state and the upper and lower polariton states follows the here decreasing, single molecule Rabi splitting, which results in a slightly different dynamics for different *N*.^24^

**Fig. 5:**
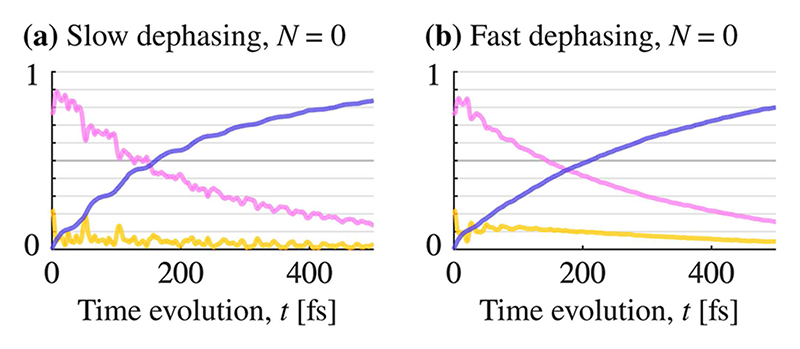
Population time-evolution of the polaritonic potential energy surfaces is shown in Fig. 3(a). The colors match the ones used for the surfaces in Fig. 3. In the left column (a), the dephasing is slow (*γ* ≈ 0.002 fs^−1^), and in the right column (b), dephasing is fast (*γ* ≈ 0.09 fs^−1^). The left gray arrows in Fig. 4 show the data point for (a), and the right gray arrow is the data point for (b). The two cases, (a) and (b), behave very similarly, the most apparent difference is the dampening of interference for fast dephasing, which makes the curves in (b) smoother.

With no two-level systems, i.e., *N* = 0, the impact of dephasing on the energy retention is small (black curve in Fig. 4). Figures 5(a) and 5(b) compare the populations in the polaritonic basis [from Fig. 3(a)] for a slow and fast dephasing rate (and *N* = 0). The most obvious difference in the populations is the amount of oscillations caused by interference and by the avoided crossing in polaritonic states. We can understand the dampened oscillations as dephasing canceling the otherwise coherent population transfer between different trajectories.

**Fig. 6:**
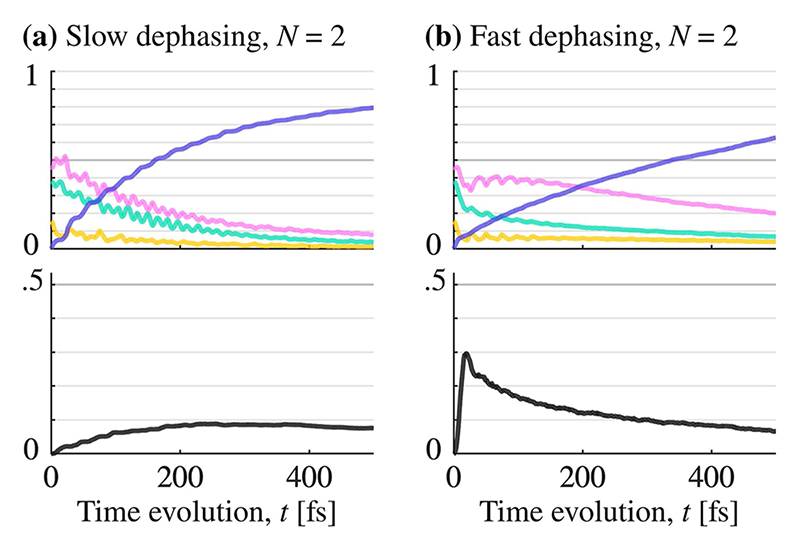
Population time-evolution of the polaritonic potential energy surfaces is shown in Fig. 3(b). The colors match the ones used for the surfaces in Fig. 3. In the left column (a), the dephasing is slow (*γ* ≈ 0.002 fs^−1^), and in the right column (b), dephasing is fast (*γ* ≈ 0.09 fs^−1^). The left blue arrows in Fig. 4 show the data point for (a), and the blue arrow is the data point for (b).

Introducing dark states (*N* ≥ 2) will make energy retention increasingly sensitive to changes in the dephasing rate. We consider, first, the case with a single dark state (*N* = 2). Here, dephasing has an observable effect on energy retention. Figure 3(b) shows the polaritonic energy surfaces for *N* = 2, which includes a single dark state (black curve) and middle polariton state (green curve). The corresponding population evolution is shown in Fig. 6, for slow dephasing (a) and fast dephasing (b). Take note of how a fast dephasing rate imposes a rapid build-up of the population in the dark state, which peaks at about 20 fs and slowly decays thereafter. This is in contrast to the slow dephasing rate, where the dark state population occurs slowly over a timescale of ≈300 fs.

**Fig. 7:**
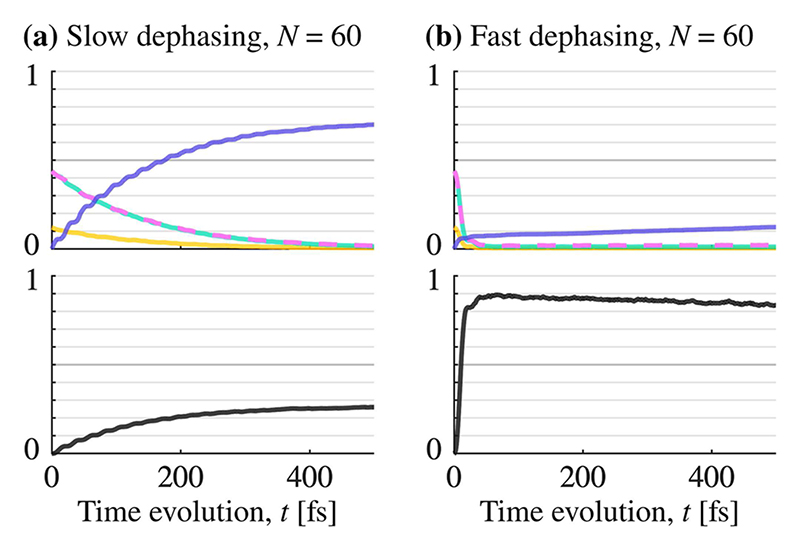
Population time-evolution of the polaritonic potential energy surfaces for *N* = 60 (which are very similar to the ones shown in Fig. 3). Colors match the ones used for the surfaces in Fig. 3(b), but the black curve is a sum of all 59 dark states. In the left column (a), the dephasing is slow (*γ* ≈ 0.002 fs^−1^), and in the right column (b), the dephasing is fast (*γ* ≈ 0.09 fs^−1^). The left yellow arrows in Fig. 4 show the data point for (a), and the right yellow arrow is the data point for (b).

The highest energy retention is observed for the largest number of two-level systems studied (*N* = 60) and a dephasing rate on the order of *γ* ≈ 10^−1^ fs^−1^. Here, the solid yellow curve in Fig. 4 shows a sharp increase in energy retention with increasing dephasing, from around 10% to almost 90%. The time-evolution of the populations for *N* = 60 is shown in Fig. 7, for both slow dephasing in (a) and fast dephasing in (b). The populations of all 59 dark states are shown as a sum (black curve). For the fast dephasing rate, the dark states population builds up rapidly and reaches its maximum value of 90% in about 40 fs. Thereafter, the dark states population is very slowly released back to the bright states, with a retention of 80% at 500 fs. For *N* = 60 and a slow dephasing rate, the build up to about 27% is slow and does not reach a maximum within 500 fs. For both slow and fast dephasing, the population in the dark states has significantly increased when compared to the case *N* = 2 (compare Figs. 6 and 7).

The mechanism for energy retention can be explained by the transition dipole moments of the collective system. The main energy loss mechanism is the decay of photons due to imperfect cavity mirrors. However, the dark states have no transition dipole moment in the absence of dephasing, as they represent asymmetric superpositions of matter excitations. Thus, the dark states do not couple to the cavity mode in an ideal system and cannot scatter photons into the cavity mode. Population in the dark states are thus protected from photon decay. However, the dephasing breaks the symmetry in the Hamiltonian that decouples the dark states from the upper, middle, and lower polariton states and enables population transfer. With an increasing number of two-level systems *N*, the number of available dark states also increases, and thus, the effective rate increases with which these states are populated. The dark state subspace can thus serve as a reservoir that protects the systems from photon decay.^49,86^

The population transfer between dark states and bright states goes both ways. As can be seen in Figs. 6(b) and 7(b), dephasing will also slowly return the population to the bright states. However, this process is slower than population transfer into dark states, resulting in an overall slowdown of energy loss.

The energy retention in Fig. 4 has a local maximum with respect to the dephasing rate at about *γ* ≈ 10^−1^ fs^−1^ and *N* = 60. However, if the dephasing rate is further increased, energy retention decreases. We interpret these phenomena based on the line-widths and Rabi-splittings: at the maximum of the energy retention, the collective Rabi-frequency (Ω_*R*_ = 2.85 × 10^−1^ fs^−1^) is in the same order as the dephasing rate (Ω_*R*_ ≈ *γ*). Thus, the Rabi oscillations (Ω_*R*_ = 2.85 × 10^−1^ fs^−1^) are dampened but not yet over dampened. If the dephasing rate is further increased, the line-width of the dark becomes large enough for the polariton states and the dark states to overlap (see scheme in Fig. 1). In this over dampened regime, the dark states are no longer sufficiently decoupled from the bright states, and there is a significant leakage from the dark states. This spectral overlap allows for a direct photon decay from the dark states. Note that this regime can also be interpreted as a weak coupling regime since the dephasing rates become larger than the Rabi-frequency.

## V Conclusion

In summary, we have investigated a molecular Tavis–Cummings model under the influence of dephasing and photon decay. The model system consisting of a CO molecule with a varying number of resonantly coupled two-level systems has been solved with a quantum trajectory approach instead of a direct solution of the Lindblad equation. This approach allowed us to use a wave function base calculation, which scales more favorably with respect to the number of states than evaluating a density matrix explicitly.

In this atomistic model, we could show that the dark states become increasingly coupled to the polariton states as the dephasing rate is increased. As a result, the population gets trapped in the dark states, and it is protected from photon decay processes. Our findings are in line with earlier studies.^49,86^ Under the influence of dephasing, the dark states are no longer decoupled from the polariton states. The effective transfer rate into the dark states even increases with an increasing number of dark states, thus providing increasingly efficient protection against photon decay. In the investigated systems, the photonic excitation was rapidly transferred into the dark state reservoir and slowly released back into the bright polariton states.

Our results show that dephasing, as it would occur under experimental conditions in a condensed phase, plays a significant role in the dynamics of such a system. Realistic models should thus not only include photon decay, which has been demonstrated to play a crucial role,^24^ but should also include the effects from pure dephasing in the condensed phase. Since the dephasing rates should depend on the temperature of the system, such an effect may be observable through temperature dependent measurements of, for example, fluorescence lifetimes.

## Supplementary Material

Appendix

## Data Availability

The data that support the findings of this study are available from the corresponding author upon reasonable request.
