# Supplementary material for: The role of dephasing for dark state coupling in a molecular Tavis–Cummings model
Source: J Chem Phys. Author manuscript; Available in PMC 2024 Feb 16. (PMC7615654; doi:10.1063/5.0155302)
Supplement: Appendix [file EMS193998-supplement-Appendix.pdf]

APPENDIX: IMPACTS FROM PHENOMENOLOGICAL DEPHASING OPERATORS

For a single two-level system, the dephasing operator in Eq. (9) has the expected effect to decay away phase information without moving the population between states. However, when a system is built from many strongly coupled sub-systems (such as in our case), this operator will include some unintended amount of driving and decay. To probe how significant this effect is, we can fully diagonalize the Hamiltonian from Eq. (2) and apply the basis transformation to a dephasing operator. (Note that we can omit the ground-state from the diagonalization since it does not couple to any other state.) This basis transformation into a fully diagonalized Hamiltonian in terms of vibrational, electronic, and photonic states will give a set of eigenstates containing some unphysical (unbound) states when the energy of the vibrational motion is enough to dissociate the molecule. However, since our time-evolution does not exhibit dissociation, we do not populate such questionable states, and they could be excluded from this analysis without affecting the conclusion.

A dephasing operator (for $N = 2$ atoms) after transformation to the energy ordered eigenbasis is shown in Fig. 8. Driving and decay will appear as off-diagonal elements.

On close inspection, we can see that there are off-diagonal elements but that the matrix is essentially block diagonal. This means that there are some driving and decay between eigenstates that are close to degeneracy, but the population is trapped within each block of states. Thus, we cannot drive or decay the population further than between a few energetically adjacent states. This is not of concern to our investigation, and a phenomenological operator is

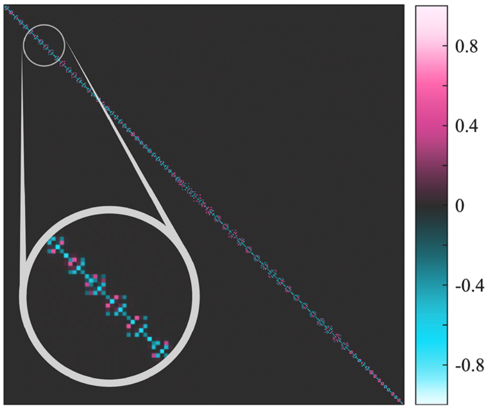

FIG. 8. Dephasing operator after transformation to eigen-basis. The initially diagonal operator acquires off-diagonal elements, but the operator remains block-diagonal. The 5×5 blocks represent the electronic–photonic subspace, but neighboring nuclear configurations are not coupled here.

15 August 2023 08:18:46

© Author(s) 2023

considered sufficient. For further discussions, see, for instance, Refs. 49, 87, and 88.

15 August 2023 08:18:46

© Author(s) 2023
